# Crusted Scabies Infection in the Setting of Chronic Steroid and Omalizumab Use

**DOI:** 10.7759/cureus.16490

**Published:** 2021-07-19

**Authors:** Chelsea Karson, Seetharam Mannem, Logan Morin, Lindsay Karson, Mark Rizko

**Affiliations:** 1 Psychiatry, Advocate Lutheran General Hospital, Park Ridge, USA; 2 Internal Medicine, Advocate Lutheran General Hospital, Park Ridge, USA; 3 Internal Medicine, Chicago College of Osteopathic Medicine, Midwestern University, Downers Grove, USA; 4 Internal Medicine, Chicago Medical School, Rosalind Franklin University of Medicine and Science, North Chicago, USA; 5 Internal Medicine, College of Medicine, University of Illinois, Chicago, USA

**Keywords:** crusted scabies, scabies, drug-induced itp, itp, steroid use, immunocompromised patient

## Abstract

Scabies infection is a very common skin disease that occurs due to infestation with the *Sarcoptes scabei* mite. Typically, it results in intensely pruritic papules and excoriations in the webs of the hand, groin, or axilla, and remains limited in its spread. In rare cases, the disease can become diffuse and progress to crusted or nodular subtypes. Here, we report the case of crusted scabies infestation in a 69-year-old male who presented with a diffuse pruritic, erythematous, and petechial rash. His medical history was significant for severe idiopathic urticaria treated with omalizumab. Before starting omalizumab, the patient was self-medicating for several months with corticosteroids obtained through his veterinary practice to alleviate symptoms. His presentation was complicated by immune thrombocytopenic purpura and muscle weakness, likely secondary to omalizumab and corticosteroid use, respectively. The patient underwent an extensive rheumatologic workup until skin biopsy confirmed the underlying etiology as crusted scabies infestation. He was treated with ivermectin and weekly 5% permethrin skin cream with great improvement of his rash; however, unfortunately, he succumbed to bacterial sepsis. Scabies infestation can masquerade as a manifestation of other systemic diseases and is often misdiagnosed. As this case illustrates, initial misdiagnosis and subsequent treatment with immunosuppressive drug regimens can cause preventable, but potentially fatal, concomitant superinfections.

## Introduction

Scabies is an infestation of the skin by the mite *Sarcoptes scabei*. It is estimated to infect up to 100 million people worldwide, and its risk factors include poverty and crowding [1,2]. Scabies is typically transmitted through prolonged skin exposure to an infested patient, often among those living together [3]. Female mites burrow into the skin and deposit eggs and feces, which trigger a delayed (type IV) hypersensitivity reaction that leads to the classic pruritic rash [4].

In the classic form of scabies, patients present with mite burrows, papules, and excoriations in multiple locations, including the webs of fingers, wrists, axillae, areolae, and genitalia. Symptoms appear three to six weeks after exposure, with most cases involving 10-15 mites per patient [5]. In addition to intense pruritus that affects the patient’s quality of life, scabies excoriations can predispose to local cutaneous infections such as cellulitis or systemic infections. Symptoms typically worsen at night, with insomnia and other psychological sequelae [6]. Ivermectin is a highly effective treatment for scabies that works by acting on the muscle cells and neurons of mites, which paralyzes them [6]. Permethrin is another treatment option that works in a similar fashion [7].

A less common but more severe presentation is crusted scabies, which typically manifests in immunocompromised hosts [8]. However, in up to 40% of cases, patients with crusted scabies are not immunocompromised and do not have any identifiable risk factors, suggesting a possible genetic etiology [9]. Crusted scabies involves scales, crusts, and fissures that may encompass all skin surfaces [8]. Hosts may be infested with millions of mites, and transmission can occur through limited skin contact and fomites [10]. Therefore, crusted scabies poses an especially high transmission risk, and healthcare workers must take appropriate precautions when caring for these patients.

## Case presentation

**Figure 1:**
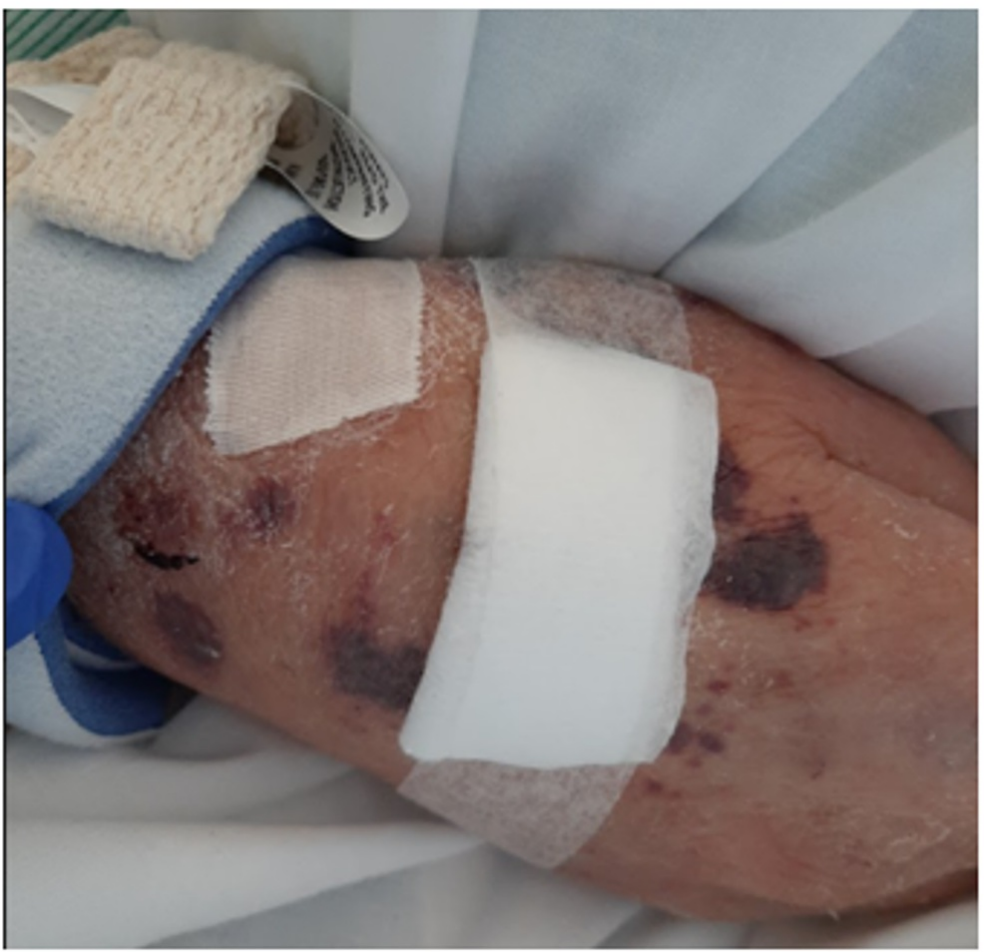
Patient’s right hand with visible white scaling and ecchymosis.

**Figure 2:**
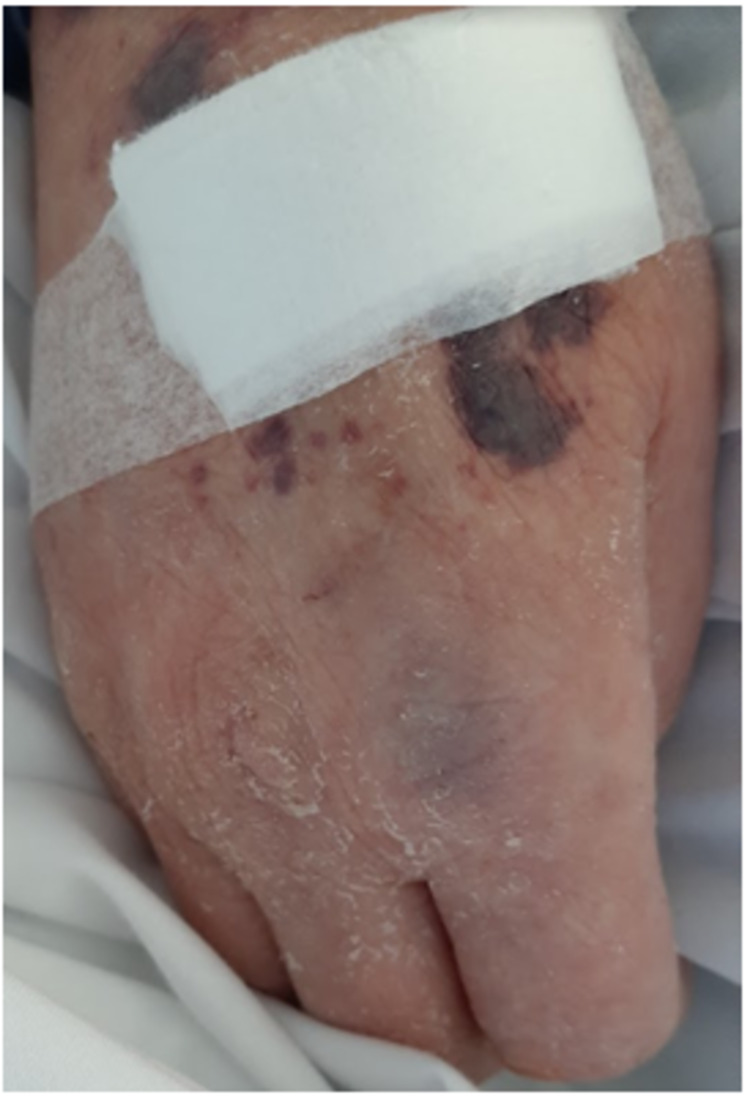
Alternate view of the patient’s right hand showing scaling and ecchymosis.

A 69-year-old male veterinarian with a past medical history of anxiety, depression, and idiopathic urticaria presented to the emergency department complaining of a six-month history of a diffuse, intensely pruritic rash and a two-month history of rapidly progressing muscle weakness. The rash began after a trip to Mexico; he did not recall any sick contacts. The rash was notably more intense than his chronic urticaria and failed to respond to antihistamines. It initially appeared in the groin but spread diffusely. The patient attempted to self-medicate by self-prescribing oral corticosteroids periodically for several months. After the self-prescribed corticosteroids failed to relieve his symptoms, he sought care with an outpatient rheumatologist who started the patient on omalizumab with minimal improvement. Over the course of his treatment, he developed weakness of his proximal muscles and became dependent on a walker for ambulation. His symptoms were disturbing his work and sleep schedule. He reported that he was recently hospitalized for lower extremity cellulitis that developed during the course of these symptoms, which was treated with trimethoprim-sulfamethoxazole. On physical examination, he had scattered petechiae, excoriations, and crusts throughout the trunk and extremities, along with scaling and ecchymoses of the hands and feet (Figures 1, 2). In the emergency department, he was found to have a white blood cell count of 8.3/nL, hemoglobin of 11.2 g/dL, a severely depressed platelet count of 17,000/nL, erythrocyte sedimentation rate of 29 mm/hour, D-dimer of 3.14 µg/mL, fibrinogen of 424 mg/dL, and antithrombin III activity of 105%. Hematology was consulted with suspicion of idiopathic versus drug-induced thrombocytopenia secondary to omalizumab use as an etiology of the scattered petechiae and ecchymoses. An extensive rheumatologic workup, including antinuclear antibody, anti-cytoplasmic antibody, and tryptase, was noncontributory. Dermatology was consulted and a skin biopsy was performed. During his hospital stay, the patient required rituximab after treatment with two platelet transfusions and intravenous immunoglobulin was unsuccessful. He was discharged with a plan for three rituximab infusions and a prednisone taper. His muscle strength improved with inpatient rehabilitation and he was discharged home on a walker, with instructions for outpatient follow-up.

The biopsy results came back after discharge showing a high density of scabies mites and ova, consistent with a crusted scabies infection. The patient was prescribed ivermectin 18 mg on days one, two, eight, nine, and fifteen as well as 5% topical permethrin cream once weekly for four weeks. Although the patient had been working at his busy practice for much of his illness, none of his colleagues reported scabies infection. This is likely because they were not immunocompromised and infestation typically requires at least 10-15 minutes of close skin-to-skin contact to transfer the mites [11,12]. He returned to the hospital after suffering a fall at home a week later, complicated by a T12 wedge compression fracture. He had not started his treatment regimen. On examination, his rash had not improved; he was weaker than before and his mental status had deteriorated. The scabies was treated with ivermectin and permethrin. Although the rash improved significantly with the treatment, his hospital course was complicated by *Staphylococcus aureus* bacteremia with an epidural abscess, which was drained. Afterward, unfortunately, his mental status progressively declined with cardiopulmonary decompensation while receiving treatment for sepsis and he passed away.

## Discussion

This case demonstrates the importance of avoiding confirmation bias with skin diseases. It is unique in demonstrating a previously unreported misdiagnosis of chronic urticaria. An early missed diagnosis likely prolonged and complicated the course of the disease in our patient. Crusted scabies infestations are commonly misdiagnosed initially as psoriasis, eczema, contact dermatitis, or severely dry skin [11]. When this occurs, patients are often treated with topical steroids which exacerbates their condition further [12].

Scabies is more commonly seen in patients of less affluent backgrounds. The diagnosis is not often associated with well-educated professionals like our patient and therefore is more likely to be missed [11]. The major risk factor demonstrated by our patient was chronic drug-induced immunosuppression. As immunosuppressive medications are becoming more commonly prescribed and immunocompromised patients enjoy longer lives, crusted scabies infestations can be expected to increase in frequency [13]. Thus, it is important to include scabies in the differential of refractory pruritus, even in nonclassic presentations.

In particular, crusted scabies infestations should be diagnosed early as they are associated with greater morbidity and mortality. When not appropriately treated, patients with crusted scabies can suffer complications such as skin infections, as seen in our case [8]. The fissures associated with crusted scabies are thought to especially predispose to bacteremic superinfection. The 30-day mortality of *S. aureus* bacteremia has been reported to be 16% [14]. A retrospective review of over 200 cases of crusted scabies in one hospital demonstrated that 11% developed a superinfection with *S. aureus* bacteremia, with a 7% 30-day mortality in this cohort [15]. A prospective analysis of 80 patients with crusted scabies demonstrated reduced mortality with early treatment with oral ivermectin and topical anti-scabicidal cream [16].

In a typical scabies infestation, extreme pruritus is caused by the activation of inflammatory cells such as lymphocytes, histiocytes, and eosinophils in response to the feces of the mites [12]. Our patient’s immune response had been suppressed by a prolonged course of oral steroids, which provided him with some relief from the pruritus, but allowed the mites to proliferate and develop into crusted scabies [11]. The steroid creams he had applied to his skin altered his immune response, repressed his inflammatory response, and suppressed his cellular immunity [7]. Thus, early misdiagnosis not only delayed appropriate treatment but resulted in a course of intervention that complicated the disease course.

Although treatment with 5% permethrin cream and ivermectin is effective, our patient had already been discharged from the hospital [17,18]. He had declined home health services and was too weak to begin treatment independently, eventually returning to the hospital after falling at home. While at the hospital, the patient received appropriate treatment and scabies resolved; however, the patient’s health declined and he succumbed to secondary *S. aureus* sepsis.

## Conclusions

This case illustrates the importance of considering scabies when treating immunocompromised patients with severe, pruritic rashes. It also stresses the importance of obtaining skin biopsy when investigating unresolved rashes that are thought to be atopic or urticarial and do not respond to conventional treatment. In this patient, delayed diagnosis slowed treatment and left him vulnerable to complications. Therefore, it is useful to obtain a pathological diagnosis in these scenarios before starting immunosuppressive therapy to prevent the worsening of latent infections.

## References

[1] Leung AK, Lam JM, Leong KF (2020). Scabies: a neglected global disease. Curr Pediatr Rev.

[2] Fuller LC (2013). Epidemiology of scabies. Curr Opin Infect Dis.

[3] Gilson RL, Crane JS (2021). Scabies. https://pubmed.ncbi.nlm.nih.gov/31335026/.

[4] Walton SF, Oprescu FI (2013). Immunology of scabies and translational outcomes: identifying the missing links. Curr Opin Infect Dis.

[5] Chosidow O (2000). Scabies and pediculosis. Lancet.

[6] Gunning K, Kiraly B, Pippitt K (2019). Lice and scabies: treatment update. Am Fam Physician.

[7] Bonazzetti C, Pagani G, Giacomelli A (2020). A case of crusted scabies with a delayed diagnosis and inadequate therapy. Infez Med.

[8] Sánchez-Borges M, González-Aveledo L, Capriles-Hulett A, Caballero-Fonseca F (2018). Scabies, crusted (Norwegian) scabies and the diagnosis of mite sensitisation. Allergol Immunopathol (Madr).

[9] Mitra M, Mahanta SK, Sen S, Ghosh C, Hati AK (1995). Transmission of Sarcoptes scabiei from animal to man and its control. J Indian Med Assoc.

[10] Karthikeyan K (2009). Crusted scabies. Indian J Dermatol Venereol Leprol.

[11] Centers for Disease Control and Prevention. (2019, October 2 (2021). Centers for Disease Control and Prevention. Scabies: medications. https://www.cdc.gov/parasites/scabies/health_professionals/meds.html.

[12] Binić I, Janković A, Jovanović D, Ljubenović M (2010). Crusted (Norwegian) scabies following systemic and topical corticosteroid therapy. J Korean Med Sci.

[13] Lima FC, Cerqueira AM, Guimarães MB, Padilha CB, Craide FH, Bombardelli M (2017). Crusted scabies due to indiscriminate use of glucocorticoid therapy in infant. An Bras Dermatol.

[14] Cartron AM, Boettler M, Chung C, Trinidad JC (2020). Crusted scabies in an elderly woman. Dermatol Online J.

[15] Wang MK, Chin-Yee B, Lo CK (2019). Crusted scabies in a renal transplant recipient treated with daily ivermectin: a case report and literature review. Transpl Infect Dis.

[16] Hasan T, Krause VL, James C, Currie BJ (2020). Crusted scabies; a 2-year prospective study from the Northern Territory of Australia. PLoS Negl Trop Dis.

[17] Salavastru CM, Chosidow O, Boffa MJ, Janier M, Tiplica GS (2017). European guideline for the management of scabies. J Eur Acad Dermatol Venereol.

[18] Agyei M, Ofori A, Tannor EK, Annan JJ, Norman BR (2020). A forgotten parasitic infestation in an immunocompromised patient-a case report of crusted scabies. Pan Afr Med J.

